# Altered Hippocampo-Cerebello-Cortical Circuit in Schizophrenia by a Spatiotemporal Consistency and Causal Connectivity Analysis

**DOI:** 10.3389/fnins.2017.00025

**Published:** 2017-01-30

**Authors:** Xi Chen, Yuchao Jiang, Lin Chen, Hui He, Li Dong, Changyue Hou, Mingjun Duan, Mi Yang, Dezhong Yao, Cheng Luo

**Affiliations:** ^1^Key Laboratory for NeuroInformation of Ministry of Education, High-Field Magnetic Resonance Brain Imaging Key Laboratory of Sichuan Province, Center for Information in Medicine, School of Life Science and Technology, University of Electronic Science and Technology of ChinaChengdu, China; ^2^Department of Psychiatry, The Fourth People's Hospital ChengduChengdu, China

**Keywords:** local consistency, granger causal analysis, schizophrenia, hippocampus, resting-state fMRI, Chinese Clinical Trial Registry, Registration number. ChiCTR-RCS-14004878.

## Abstract

In the current study, FOur-dimensional Consistency of local neural Activities (FOCA) analysis was used to investigate the local consistency by integrating the temporal and spatial information of the local region. In the current study, resting-state fMRI data of 69 schizophrenia patients and 70 healthy controls were collected. FOCA was utilized to investigate the local consistency. Moreover, Granger causal analysis was used to investigate causal functional connectivity among these areas, which exhibited significantly different local consistency between groups. Compared with the healthy controls, the schizophrenia patients exhibited increased local consistency in hippocampus, basal ganglia and cerebellum regions, and decreased local consistency in sensoriperceptual cortex. In addition, altered causal functional connectivity was observed in hippocampo–cerebello-cortical (occipital) circuit. These findings suggested that this circuit might play a role in the motor dysfunction in schizophrenia, and should be paid more attention in future.

## Introduction

Schizophrenia is a multidimensional psychotic syndrome and associates with substantial disability as well as personal and societal costs. Various neural imaging techniques have been extensively used to investigate the brain mechanisms of this disease. Resting-state functional magnetic resonance imaging (fMRI) (Biswal et al., 1995) has been used as a powerful tool for studying human brain functions and dysfunctions (Luo et al., 2012a, 2015; Cao et al., 2014; Jiang et al., 2016), especially in the schizophrenia because it often acquires good compliance (Yu et al., 2012; Robinson et al., 2015). In previous studies, several resting-state fMRI measurements, such as Regional Homogeneity (Yu et al., 2013) and Functional Connectivity Density (Chen et al., 2015), had been used to investigate the local coherence abnormalities in schizophrenia patients. Abnormalities of local coherence in the prefrontal, limbic and occipital areas were frequently found in schizophrenia patients.

However, the above-mentioned resting-state measures only focused on the temporal correlation of the voxels (temporal consistency) in the local area, whereas disregarded the effects of the regional stability of the spontaneous brain activity signal (spatial consistency) between neighboring time points. Microstate of EEG data is a transiently stable brain state. It is an index of brain stability of the brain activity signal in EEG study (Kindler et al., 2011). It can be regarded as physiologic evidence for the coordinated activity of several neuronal sets. A new brain activity pattern emerging suggests that the brain has switched to another functional state. Resting-state EEG studies found that the schizophrenia patients exhibited increased duration of brain microstates and reduced topographic variability (Stevens et al., 1997). Previous fMRI studies also identified the altered stability of cortical signal processing in schizophrenia, and this abnormality strongly correlated with psychotic symptoms (Winterer et al., 2006). Thus, the stability of brain signal may contribute to our understanding of the pathology mechanism of schizophrenia.

In the current study, a new voxelwise data-driven measure, named FOur-dimensional (spatiotemporal) Consistency of local neural Activities (FOCA) (Dong et al., 2015), was used to investigate abnormalities of local spontaneous brain activity in schizophrenia patients. The FOCA measure is proposed to characterize the local spontaneous activity consistency by integrating temporal homogeneity of local adjacent voxels and regional stability of brain activity states between adjacent time points. Clusters with high FOCA value have high local spontaneous activity consistency.

In the present study, we compared whole-brain FOCA value between 73 schizophrenia patients and 70 healthy controls in resting-state fMRI data. This analysis could provide us with the brain areas with different local spontaneous activity consistency. In addition, we wanted to explore how these areas communicate with each other. Granger causal analysis (GCA) can measure effective connectivity. A Granger causal connectivity from a region A to another region B means that the neuronal activity in A can predict the activity in B. Thus, GCA is a useful approach to identify the causal relationships that may exist between brain regions (Jiao et al., 2011). Then, brain regions with significant FOCA differences between groups were selected as seeds. GCA (Granger, 1969) was used to detect the causal connectivity between these seeds. The current study might address the following questions: What were the local spontaneous activity consistency differences between the two groups? Did the causal connectivity between the areas, which exhibited group differences of local consistency, changed in schizophrenia?

## Materials and methods

### Subjects

Seventy-three schizophrenia patients (44 males, age range 16–66 years) and seventy controls (39 males, age range 18–68 years) were recruited for this study. The exclusion criteria included a history of neurological illness, traumatic brain injury or substance-related disorders. All patients were on medication. The diagnosis of schizophrenia was confirmed by the structured clinical interview for DSM-IV Axis I disorders—clinical version (SCID-I-CV). The Positive and Negative Syndrome Scale (PANSS) was used to assess the severity of clinical symptoms of patients (Twenty patients' assessments were missing). Written informed consent was obtained from each patient and control subject. The study was approved by the Ethics Committee of Chengdu Mental Health Center in accordance with the Helsinki Declaration. Detailed participant information can be found in Table 1.

**Table 1:** **Demographic and clinical characteristics of the participants**.

**Characteristic**	**Schizophrenia**		**Healthy control**		**Significance**	
	**Mean**	***SD***	**Mean**	***SD***	*T*-value/chi-square^*^	***P*-value (two-tailed)**
Age (years)	38.91	12.47	37.16	14.58	0.76^¶^	0.45
Gender (% male)	63.8%		55.7%		0.94^§^	0.33
Education (years)	11.55	2.39	11.51	3.19	0.08^¶^	0.94
Disease duration (years)	14.06	11.03				
Medication dosage in CPZ equivalents (mg)	318.91	171.25				
PANSS-positive score	13.68	5.68				
PANSS-negative score	20.36	6.43				
PANSS-global score	27.94	5.31				
PANSS-total score	61.98	12.86				

* Two-tailed t-tests (

¶ ) and chi-square tests (

§ *) were conducted to assess group differences for continuous and discrete variables, respectively*.

*CPZ, chlorpromazine*.

### Image acquisition

Neuroimaging data were collected on a 3-Tesla MRI scanner (GE DISCOVERY MR 750, USA) in Center for Information in Medicine (CIM) of University of Electronic Science and Technology of China (UESTC). All participants were instructed to keep relax and close their eyes without falling asleep. The functional images were acquired using a gradient-echo echo-planar imaging (EPI) sequence. The scan parameters were as follows: TE/TR = 30/2000 ms; flip angle = 90°; image matrix = 64 × 64; field of view = 24 × 24 cm; slices = 35; and slice thickness = 4 mm (no gap). A total of 255 volumes were acquired over a 510 s period. The first five volumes were discarded to remove saturation effects.

### Image preprocessing

Data preprocessing was carried out utilizing SPM8 (http://www.fil.ion.ucl.ac.uk/spm/software/spm8). The preprocessing steps included slice time correction, realignment, and spatial normalization (3 × 3 × 3 mm^3^) (Dong et al., 2015). Participants were excluded from further analyses if head motion exceeded 1.5 mm or 1.5 degree during fMRI acquisition. In addition, the translation and rotation between groups were assessed by averaging the relative displacement from every time point for each subject (Van Dijk et al., 2012).

### FOCA analysis

The FOCA maps (Dong et al., 2015) of all subjects were calculated using the FOCA toolbox (http://www.neuro.uestc.edu.cn/FOCA.html). In brief, for each voxel, a FOCA value was obtained by calculating the product of the mean temporal correlation (across the neighboring 26 voxels) and the mean spatial correlation of a local region (across the neighboring time points). Before calculating FOCA maps, nuisance signals, including 6 head motion parameters, linear trends, individual mean white matter and cerebrospinal fluid signals, were removed from the unsmoothed resting-state fMRI data. Since spatial smoothing may artificially enhance temporal consistency in the local region, we didn't conduct spatial smoothing before computing FOCA. The FOCA maps were normalized via dividing by the mean value of the whole brain to reduce the effect of individual variability. The resulting mFOCA maps were spatially smoothed [6-mm full-width at half maximum (FWHM)]. Then, random-effect one-sample *t*-tests (*P* < 0.05, FWE corrected) were conducted to produce the thresholded maps. To study the differences between the FOCA maps from patients and controls, two-sample *t*-tests were performed (*P* < 0.05, FDR corrected) while controlling for age, gender and education level.

### Effective connectivity analysis

To further detect causal connectivity among the regions with FOCA differences between groups, GCA was used. GCA uses multiple linear regressions to investigate whether one time series can correctly predict another (Friston, 2011; Luo et al., 2012b). In this study, ROIs were defined by spherical regions (radius 3 mm) of significant FOCA differences, centered at the peak *T*-values. Then, the coefficient-based, first-order GCA was performed on the mean ROI signals (Chen et al., 2009; Liao et al., 2014) (REST, http://www.restfmri.net). Because signed path coefficients are considered to be normally distributed, parametric statistical analysis can be used for group-level inferences (Hamilton et al., 2011). One-sample *t*-tests were performed on each group's GCA map to determine these edges which were significantly different from zero (*P* < 0.05, FDR corrected). In addition, two-sample *t*-test was performed on the two groups' GCA maps to determine the paths that showed significant differences between groups (*P* < 0.005, uncorrected). The group-averaged weighted in- and out-degree of each ROI was computed separately. In detail, the in-degree of a node refers to the sum of the number of paths the node has projecting to itself. Out-degree of a node refers to the sum of the number of paths the node has projecting to other nodes. The in-degrees and out-degrees of the nodes were sorted in descending order to identify causal targets or causal source levels. In addition, Wilcoxon rank-sum tests were used to investigate group differences of the in-degrees and out-degrees of each ROI (*P* < 0.05, FDR corrected).

### Correlations between functional properties and clinical variables

To study the relation between the functional measures and clinical features (including disease duration and PANSS positive, negative, and general psychopathology subscales and total scores) in patients, partial correlations were performed while controlling for age, gender, education level and medication dosage.

## Results

Four patients were excluded from the analysis because of excessive head motion; there were then 69 schizophrenia patients and 70 healthy controls in this study. For the remaining subjects, there were no significant differences in mean head motion and maximum head motion between the groups. (two-sample *t*-test, *T* = −0.851, *P* = 0.396; *T* = −0.946, *P* = 0.346).

### FOCA analysis

Brain regions located in the frontal cortex, temporal gyrus, parietal cortex, posterior cingulate cortex and occipital cortex exhibited high mFOCA values in both groups (*P* < 0.05, FWE corrected) (See Figure S1). This pattern was similar to that exhibited by other local functional homogeneity measures (Zang et al., 2004; Jiang et al., 2016).

**Figure 1:**
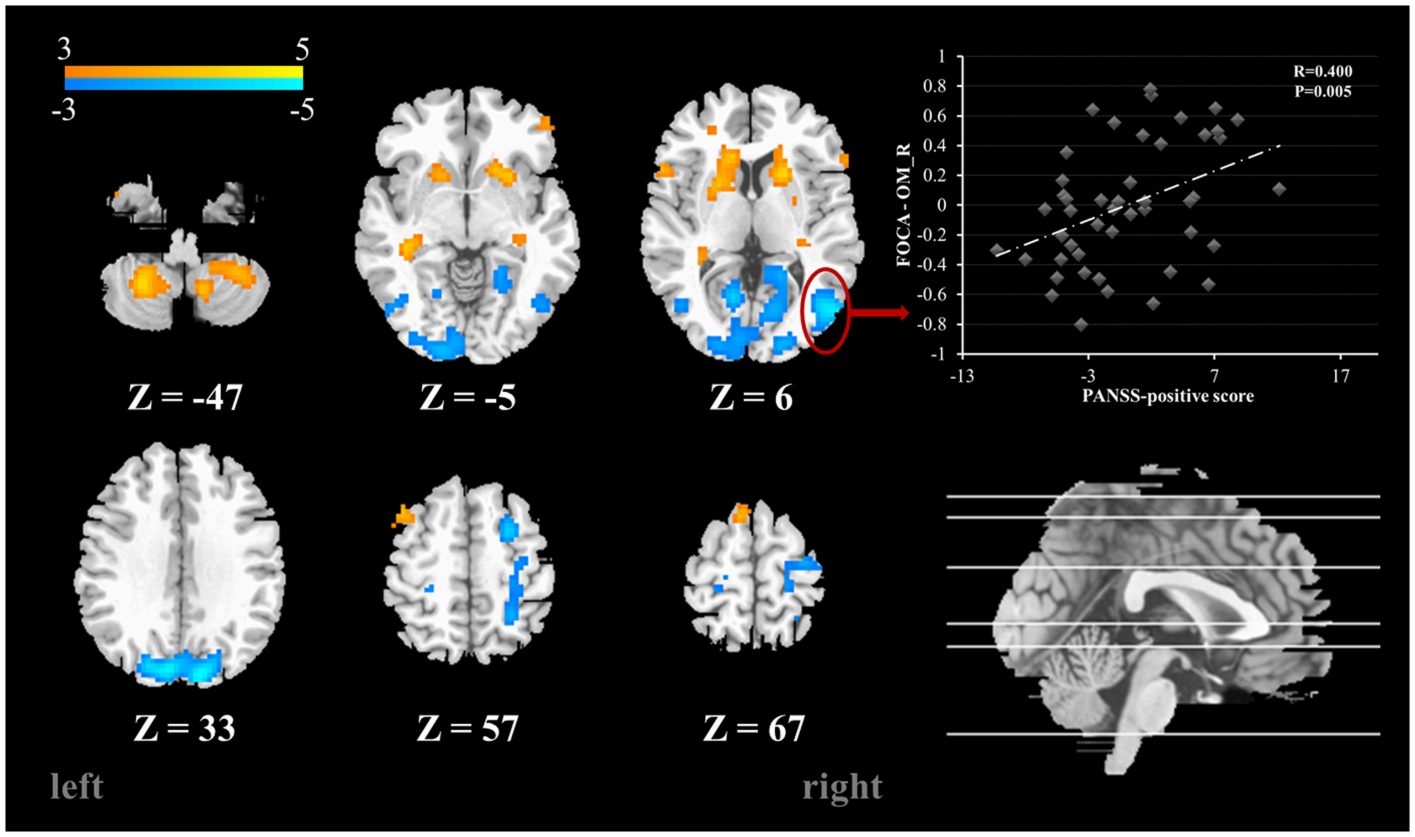
**Brain areas with significant FOCA value differences between groups (***p*** < 0.05, FDR corrected)**. Color bar represents *t*-values. Scatter plots depicting the significant positive correlations relationship between the residual of regional FOCA in the right MOG (functional properties that were regressed using controlling variables) and the residual of PANSS positive score (PANSS positive score that were regressed using controlling variables).

Compared with the healthy controls, schizophrenia patients showed higher FOCA value in the bilateral cerebellum inferior lobe, inferior temporal gyrus, putamen, caudate nucleus and hippocampus, which mean relatively high local spontaneous activity consistency in these areas. Decreased FOCA value in patients was found in the bilateral occipital cortex and postcentral gyrus (FDR corrected, *P* < 0.05) (See Table 2 and Figure 1). There were 20 clusters with markedly altered FOCA values: left middle frontal gyrus (MFG), right superior frontal gyrus (SFG), bilateral caudate nucleus (Caud), bilateral putamen (Put), bilateral hippocampus (Hip), bilateral postcentral gyrus (PoC), bilateral inferior temporal gyrus (ITG), bilateral calcarine (Cal), bilateral middle occipital gyrus (MOG), bilateral superior occipital gyrus (SOG) and bilateral inferior cerebellum lobe (IC). These areas were selected as ROIs for the subsequent effective connectivity analysis.

**Table 2:** **Summary of the FOCA differences between groups**.

**Region**	**Abbreviation**	**MNI coordinate**			***T*-value**
		**x**	**y**	**z**	
Caudate nucleus_L	Caud_L	−15	0	24	4.86
Putamen_L	Put_L	−21	6	9	4.85
Inferior cerebellum lobe_L	IC_L	−27	−57	−51	4.58
Hippocampus_L	Hip_L	−36	−33	−6	4.39
Inferior cerebellum lobe_R	IC_R	12	−63	−48	4.38
Putamen_R	Put_R	21	12	6	4.32
Inferior temporal gyrus_L	ITG_L	−45	0	−36	4.09
Caudate nucleus_R	Caud_R	18	15	6	4.06
Inferior temporal gyrus_R	ITG_R	48	3	−42	3.81
Hippocampus_R	Hip_R	32	−31	−6	3.36
Middle frontal gyrus_L	MFG_L	−42	15	54	3.34
Middle occipital gyrus_L	MOG_L	−47	−73	2	−3.18
Postcentral gyrus_L	PoC_L	−24	−36	57	−3.38
Postcentral gyrus_R	PoC_R	27	−51	54	−3.78
Superior frontal gyrus_R	SFG_R	25	6	54	−4.13
Calcarine_R	Cal_R	12	−70	13	−4.30
Calcarine_L	Cal_L	−12	−66	6	−4.42
Middle occipital gyrus_R	MOG_R	51	−75	3	−4.85
Superior occipital gyrus_L	SOG_L	−19	−88	24	−4.97
Superior occipital gyrus_R	SOG_R	12	−90	27	−5.42

*T-value reflects the peak FOCA value in the area*.

*L, left; R, right*.

### Effective connectivity analysis

**Figure 2:**
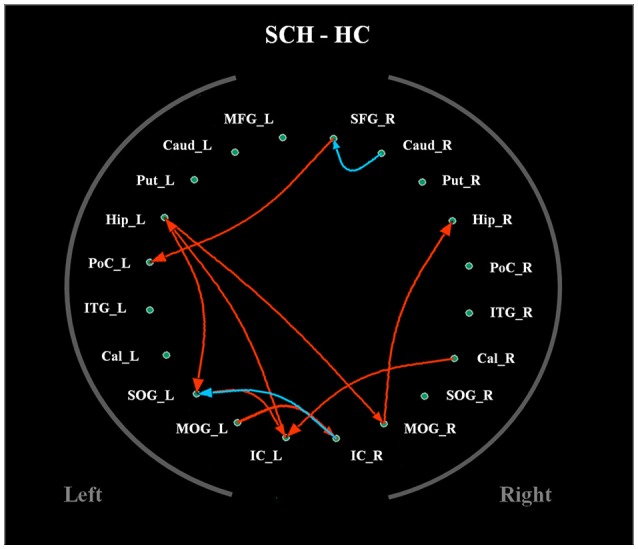
**The two-sample ***t***-test results of the effective connectivity paths among the 20 ROIs**. (*p* < 0.005, uncorrected) (|*t*| value range: 2.85–3.84) (red line: SCH > HC; blue line: HC > SCH). Abbreviations: SCH, schizophrenia; HC, healthy control.

**Figure 3:**
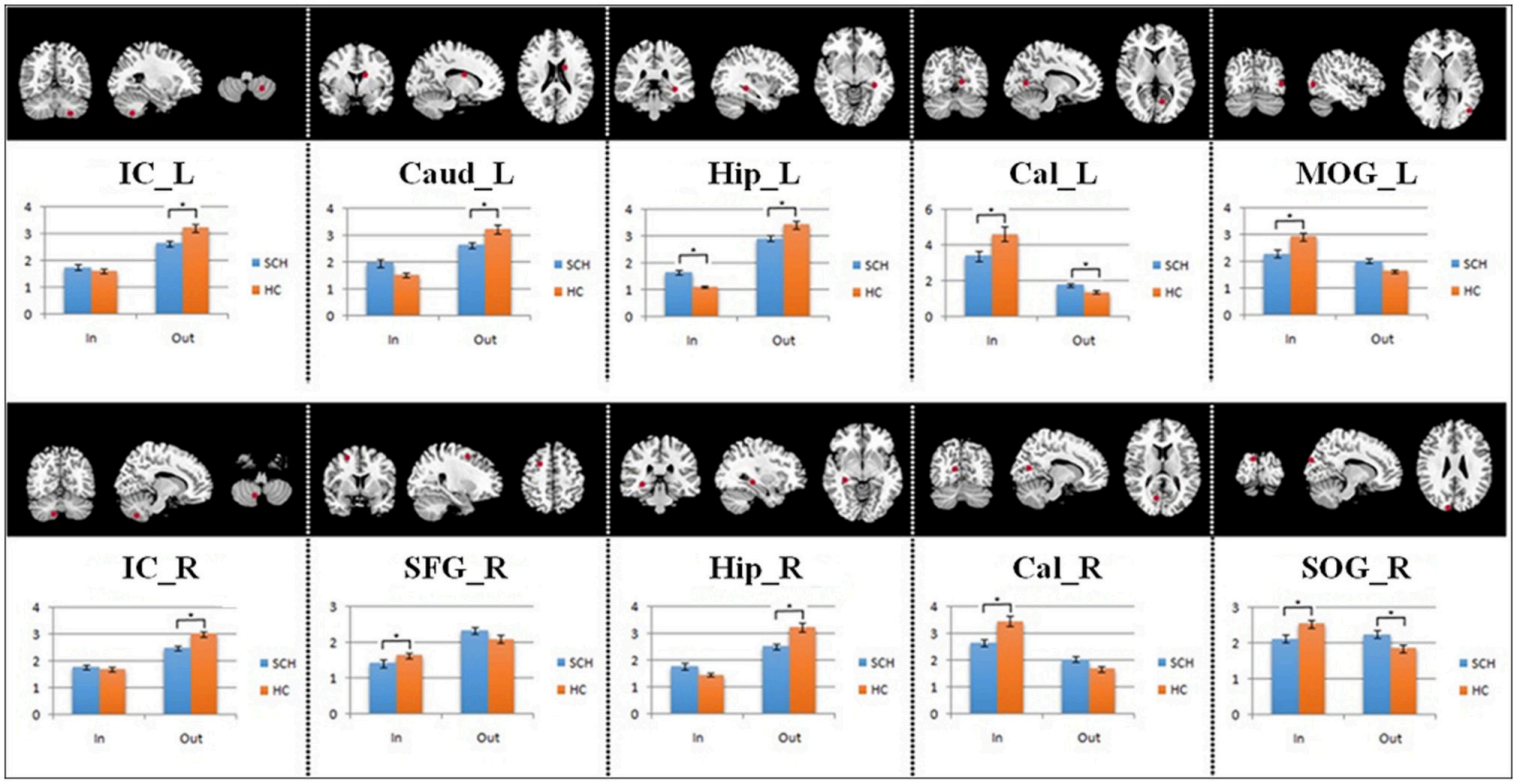
**The quantitative comparison results of in- and out-degree differences between the two groups**. The asterisk represents significant group differences (*P* < 0.05, FDR corrected).

The average Granger-causality interaction magnitudes of each ROI pair of the two groups were demonstrated in Tables S1, S2. For the one-sample *t*-test results (Figure S2), the red lines represented those connections within the left hemisphere, the blue lines represented those connections within the right hemisphere, and purple lines represented the interhemispheric connections (*P* < 0.05, FDR corrected). The patients exhibited more paths than the healthy controls. Two-sample *t*-test found that the patients had 8 increased paths (Figure 2) (*P* < 0.005, uncorrected) (red lines). Three of the paths were among regions in the left hemisphere (Hip → SOG, SOG → IC and IC → Hip). One path was among regions in the right hemisphere (MOG → Hip). In addition, two paths were projections from the left nodes to the right nodes (left Hip → right MOG and left MOG → right IC); two paths were projections from the right nodes to the left nodes (right SFG → left PoC and right Cal → left IC). Moreover, patients showed two decreased paths (blue lines) (right Caud → right SFG and right IC → left SOG). The main results demonstrated that the causal connections in the Hip, occipital lobe and cerebellum were increased in patients. The group-averaged in- and out-degrees of the nodes were sorted in descending order and shown in Table 3 for each group. The main outflow regions in both groups were the Hip and the striatum (Put, Caud). The occipital area was the dominant causal inflow region in both groups. The brain regions that with significant in- or out-degree group differences were shown in Figure 3. In general, the degree properties in Hip, occipital cortex and cerebellum exhibited significant group differences (Table 4).

**Table 3:** **The degree properties of the 20 ROIs of two groups**.

	**Schizophrenia**				**Healthy control**			
	**In-degree**		**Out-degree**		**In-degree**		**Out-degree**	
	**Node**	**Mean (SE)**	**Node**	**Mean (SE)**	**Node**	**Mean (SE)**	**Node**	**Mean (SE)**
1	Cal_L	3.38 (0.27)	Put_L	3.12 (0.13)	Cal_L	4.62 (0.39)	Hip_L	3.43 (0.14)
2	MFG_L	2.96 (0.20)	PoC_L	3.08 (0.12)	Cal_R	3.46 (0.17)	Put_L	3.34 (0.13)
3	MOG_R	2.66 (0.16)	Hip_L	2.92 (0.12)	MOG_R	3.21 (0.21)	Caud_L	3.23 (0.15)
4	Cal_R	2.65 (0.14)	PoC_R	2.77 (0.14)	MOG_L	2.93 (0.14)	Hip_R	3.22 (0.16)
5	ITG_R	2.32 (0.13)	Put_R	2.74 (0.10)	SOG_R	2.54 (0.10)	IC_L	3.22 (0.14)
6	ITG_L	2.30 (0.19)	SOG_L	2.65 (0.12)	MFG_L	2.47 (0.12)	PoC_L	3.11 (0.13)
7	MOG_L	2.29 (0.14)	Caud_L	2.63 (0.12)	ITG_L	2.40 (0.16)	Put_R	3.06 (0.13)
8	SOG_R	2.12 (0.11)	IC_L	2.63 (0.10)	SOG_L	2.15 (0.10)	IC_R	3.02 (0.11)
9	Caud_L	1.97 (0.15)	Caud_R	2.55 (0.11)	ITG_R	2.10 (0.16)	Caud_R	2.92 (0.12)
10	SOG_L	1.89 (0.11)	Hip_R	2.51 (0.10)	PoC_R	1.73 (0.09)	PoC_R	2.59 (0.13)
11	Hip_R	1.79 (0.12)	IC_R	2.48 (0.10)	IC_R	1.69 (0.09)	SOG_L	2.35 (0.12)
12	IC_R	1.78 (0.09)	ITG_L	2.38 (0.13)	SFG_R	1.63 (0.07)	ITG_R	2.30 (0.12)
13	IC_L	1.75 (0.11)	SFG_R	2.34 (0.10)	IC_L	1.61 (0.09)	ITG_L	2.28 (0.11)
14	Caud_R	1.66 (0.07)	SOG_R	2.25 (0.11)	Caud_R	1.52 (0.05)	SFG_R	2.09 (0.11)
15	Hip_L	1.64 (0.09)	Cal_R	2.04 (0.10)	Caud_L	1.52 (0.10)	SOG_R	1.85 (0.12)
16	Put_R	1.47 (0.05)	MOG_L	2.01 (0.09)	Hip_R	1.46 (0.06)	MFG_L	1.80 (0.09)
17	SFG_R	1.42 (0.11)	ITG_R	2.00 (0.09)	PoC_L	1.41 (0.07)	Cal_R	1.68 (0.09)
18	PoC_R	1.42 (0.07)	MOG_R	1.91 (0.09)	Put_R	1.34 (0.04)	MOG_L	1.65 (0.07)
19	Put_L	1.31 (0.05)	Cal_L	1.78 (0.09)	Put_L	1.28 (0.05)	MOG_R	1.62 (0.07)
20	PoC_L	1.16 (0.05)	MFG_L	1.57 (0.08)	Hip_L	1.16 (0.05)	Cal_L	1.38 (0.07)

*L, left; R, right*.

**Table 4:** **Two-sample ***t***-test of the GCA connectivity strength in patients and controls**.

**From**	**To**	***T*-value**	**Connectivity strength in Schizophrenia (Mean ± SE)**	**Connectivity strength in Healthy control (Mean ± SE)**
MOG_L	IC_R	3.824	0.044061 ± 0.013852	−0.063853 ± 0.024235
MOG_R	Hip_R	3.504	−0.010701 ± 0.020487	−0.12901 ± 0.026459
Cal_R	IC_L	3.465	0.023828 ± 0.0191	−0.11638 ± 0.035165
SFG_R	PoC_L	3.273	−0.00091981 ± 0.016529	−0.073819 ± 0.01471
IC_L	Hip_L	3.262	0.035209 ± 0.018883	−0.042392 ± 0.014264
SOG_L	IC_L	3.228	0.041486 ± 0.015196	−0.038431 ± 0.019267
Hip_L	MOG_R	3.124	0.031274 ± 0.0083653	0.0014383 ± 0.0045396
Hip_L	SOG_L	2.979	0.050465 ± 0.013611	0.0050824 ± 0.0067386
Caud_R	SFG_R	−3.029	0.0016412 ± 0.010733	0.043918 ± 0.0087939
IC_R	SOG_L	−3.093	−0.00054505 ± 0.01181	0.047759 ± 0.010068

*L, left; R, right*.

### Correlations between brain functional properties and clinical variables

To explore the relationships between the FOCA values and the clinical features (disease duration and PANSS positive, negative and general psychopathology subscales and total scores), partial correlations were performed. The results showed that the FOCA value of the right MOG had a significant positive correlation with PANSS positive score. The results were presented in Figure 1. There was no significant relationship between the effective connectivity path strengths and the clinical features.

## Discussion

We used FOCA to analysis the local consistency. Local functional brain consistency contains two aspects: temporal homogeneity of local adjacent voxels and regional stability of brain activity states between adjacent time points. The local coherence of a region in the temporal domain can give us information about the consistency of temporal fluctuations in local regions. The local coherence of a region in the spatial domain can give us knowledge about the regional stability of brain activity states. The region with high FOCA value means that this region has low level of temporal fluctuations and high regional stability. In the current study, we found that the schizophrenia patients exhibited higher local consistency in the frontal area, basal ganglia, Hip and cerebellum than the healthy controls; however, in the occipital cortex and bilateral postcentral gyrus, the patients showed decreased local spatiotemporal consistency. In addition, the causal connections of the Hip, occipital lobe and cerebellum were mainly increased in patients.

### Altered local consistency in schizophrenia

We identified altered local spatiotemporal functional consistency in the frontal area, basal ganglia, Hip, occipital area and cerebellum. In previous research, these areas often exhibited volumetric reduction or local temporal functional consistency alterations, even in drug naïve patients (Cordon et al., 2015; Dazzan et al., 2015; Xu et al., 2015). In the human brain, distinct cortical areas and the cerebellum are linked to specific thalamic areas via the basal ganglia (Cropley et al., 2006; Woodward et al., 2012). In addition, the limbic area, especially the Hip, exhibits intimate connections with the thalamus and the basal ganglia (Bland, 2004). Thus, we could organize these brain regions, which exhibited significantly altered spatiotemporal functional consistency, as the hippocampo–cerebello-cortical circuit. The results of node degree analysis showed that the main outflow regions in both groups were the Hip and the basal ganglia. These results suggested that the limbic area and basal ganglia were transport centers in the brain. Dysfunction of these areas might alter their impact on other brain regions, thereby caused a wide range of functional abnormality. The occipital cortex and cerebellum exhibited high spatiotemporal consistency and causal connection alterations in the patients in the current study. The functional alteration of these areas might serve as a potential basis for the deficits observed in early-stage visual processing and varied cerebellar dependent motor deficits in schizophrenia (Butler et al., 2008; Walther and Strik, 2012). Specifically, the middle occipital gyrus is an important hub of sensorimotor signal integration (Renier et al., 2010). Since basic sensorimotor information forms our perception and memory of the world around us, the alteration of these areas could be associated with the perception confusion and delusion of schizophrenia. This speculation was supported by the positive correlation between local consistency in the right middle occipital gyrus and the PANSS positive scores in patients. The PANSS positive score represented severity of the positive symptoms which did not exist in healthy controls. The function reflected by positive score would not be included in healthy controls. The reduced FOCA value in the right middle occipital gyrus in schizophrenia might suggest the impaired function in patients. Although the enhanced amplitude of FOCA in right middle occipital gyrus was closer to that in healthy controls, the higher FOCA value in schizophrenia patients might represented the more severe impairment of positive symptoms.

### Altered causal connections in schizophrenia

The increased causal connections of the bilateral Hip, occipital lobe and cerebellum were the principal functional connectivity findings of the current study (Table 4). There are evidences that the Hip is involved in mechanisms underlying sensorimotor integration (Bland and Oddie, 2001). According to this model, the Hip plays a role in the voluntary motor systems. As movement continues, the relevant sensory and movement inputs continuously ascend back to the Hip and are integrated there. Thus, there is reciprocal connections between the Hip and sensorimotor areas. This process is necessary for the initiation and maintenance of voluntary motor behavior (Hallworth and Bland, 2004). In the current study, causal connection results identified that the Hip were the main outflow regions in both groups. The Hip in schizophrenia had stronger connections with occipital lobe and cerebellum than healthy control. The causal connections between the occipital lobe and cerebellum also increased in patients. These alterations might underlie the motor dysfunction in schizophrenia (Martinelli et al., 2016).

The current study had certain limitations. First, the age range (18–66 years) of the patients in the study was wide; in addition, most patients in our group were chronic and medicated patients, which might introduce confounding effects. Second, the cognitive assessment was not included in this study. Thus, the relationship between causal connections and cognitive function could not be determined. In addition, the brain structure may differ in schizophrenia. The regional changes in gray matter volume may affect the regional homogeneity. Moreover, FOCA is a new method; although FOCA was useful for detecting resting-state and event-related MRI features, its effectiveness needs further verification (Dong et al., 2015). Finally, the rationality of the usage of GCA at the neuronal level in resting-state brain networks is still controversial. In future work, high temporal resolution fMRI (short TR) should be applied for the analysis of effective connectivity.

In conclusion, the present study was the first to examine altered local consistency by integrating the temporal and spatial information of the local region in schizophrenia patients. We observed altered local consistency in the hippocampo-cerebello-cortical circuit and altered causal functional connectivity in these areas in schizophrenia patients. Based on the results of our investigation, we suggested that the importance of Hip and sensorimotor areas should be considered in the pathophysiology of motor dysfunction in schizophrenia. These system might be paid more attention in antipsychotic treatment.

## Author contributions

XC, YJ, LC, and HH had made a substantial contribution to the conception and design the experiment and drafting and revising the article, then they gave final approval of the version to be published; LD and CH had made a substantial contribution to the analysis and interpretation of the data, and revising the article critically, and then he gave final approval of the version to be published; MD, MY, DY, and CL had made a substantial contribution to the acquisition and interpretation of the data, then they gave final approval of the version to be published.

### Conflict of interest statement

The authors declare that the research was conducted in the absence of any commercial or financial relationships that could be construed as a potential conflict of interest.

## Abbreviations

fMRI: functional magnetic resonance imaging
FOCA: FOur-dimensional Consistency of local neural Activities
GCA: Granger causal analysis
PANSS: Positive and Negative Symptom Scale
ROI: region of interesting.
