# Supplementary material for: Altered Hippocampo-Cerebello-Cortical Circuit in Schizophrenia by a Spatiotemporal Consistency and Causal Connectivity Analysis
Source: Front Neurosci. 2017 Jan 30;11:25. doi: 10.3389/fnins.2017.00025 (PMC5277003; doi:10.3389/fnins.2017.00025)
Supplement: Supplementary file 1 [file Presentation1.PDF]

Supplementary Material

Altered hippocampo-cerebello-cortical circuit in schizophrenia by a spatiotemporal consistency and causal connectivity analysis

Xi Chen[1], Yuchan Jiang[1], Lin Chen[1], Hui He[1], Li Dong[1], Changyue Hou[1], Mingjun Duan[1,2], Mi Yang[1,2], Dezhong Yao[1]*, Cheng Luo[1]*

[1] Key Laboratory for NeuroInformation of Ministry of Education, Center for Information in Medicine, High-Field Magnetic Resonance Brain Imaging Key Laboratory of Sichuan Province, School of Life Science and Technology, University of Electronic Science and Technology of China, Chengdu, China
[2] Department of psychiatry, The Fourth People's Hospital Chengdu, Chengdu, China

***Correspondence:** Corresponding to Cheng Luo and Dezhong Yao, University of Electronic Science and Technology of China, Second North Jianshe Road, Chengdu, 610054, China

E-mail: chengluo@uestc.edu.cn (C. Luo), dyao@uestc.edu.cn (D. Yao).

Supplementary method

Gray matter volume analysis

Since gray matter differences between groups may confound the results, we conducted a standard voxel-based morphometry analysis (VBM) on the structural data of patients and controls. So that the brain areas regional gray matter volumes which with significant gray matter volume group differences can be covaried out in the subsequent analysis.

Supplementary result

1. Gray matter volume analysis

We compared whole brain gray matter volume between groups. There was no significant gray matter volume difference between groups (FDR corrected, $p < 0.05$). Thus, the group differences of FOCA value may mainly due to the functional property group differences rather than the brain structure group differences.

2. Supplementary figures

Figure S1.

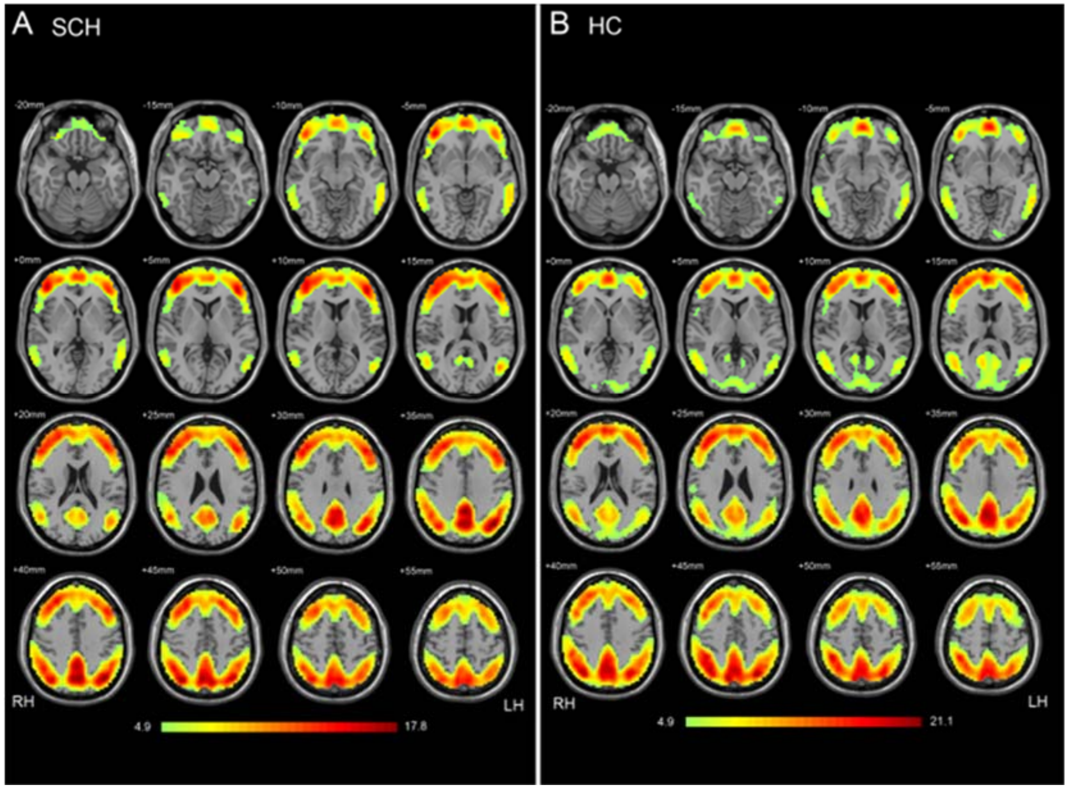

Figure S1. FOCA value distribution in the two groups. Color bar represents t values. (A) Group-level T-statistic maps showing significantly detectable mFOCA across the whole brain for schizophrenia patients (P<0.05, FWE corrected). (B) Group-level T-statistic maps showing significantly detectable mFOCA across the whole brain for health controls (P<0.05, FWE corrected).

Abbreviations: SCH=schizophrenia; HC=healthy control; RH=right hemisphere; LH=left hemisphere.

Figure S2.

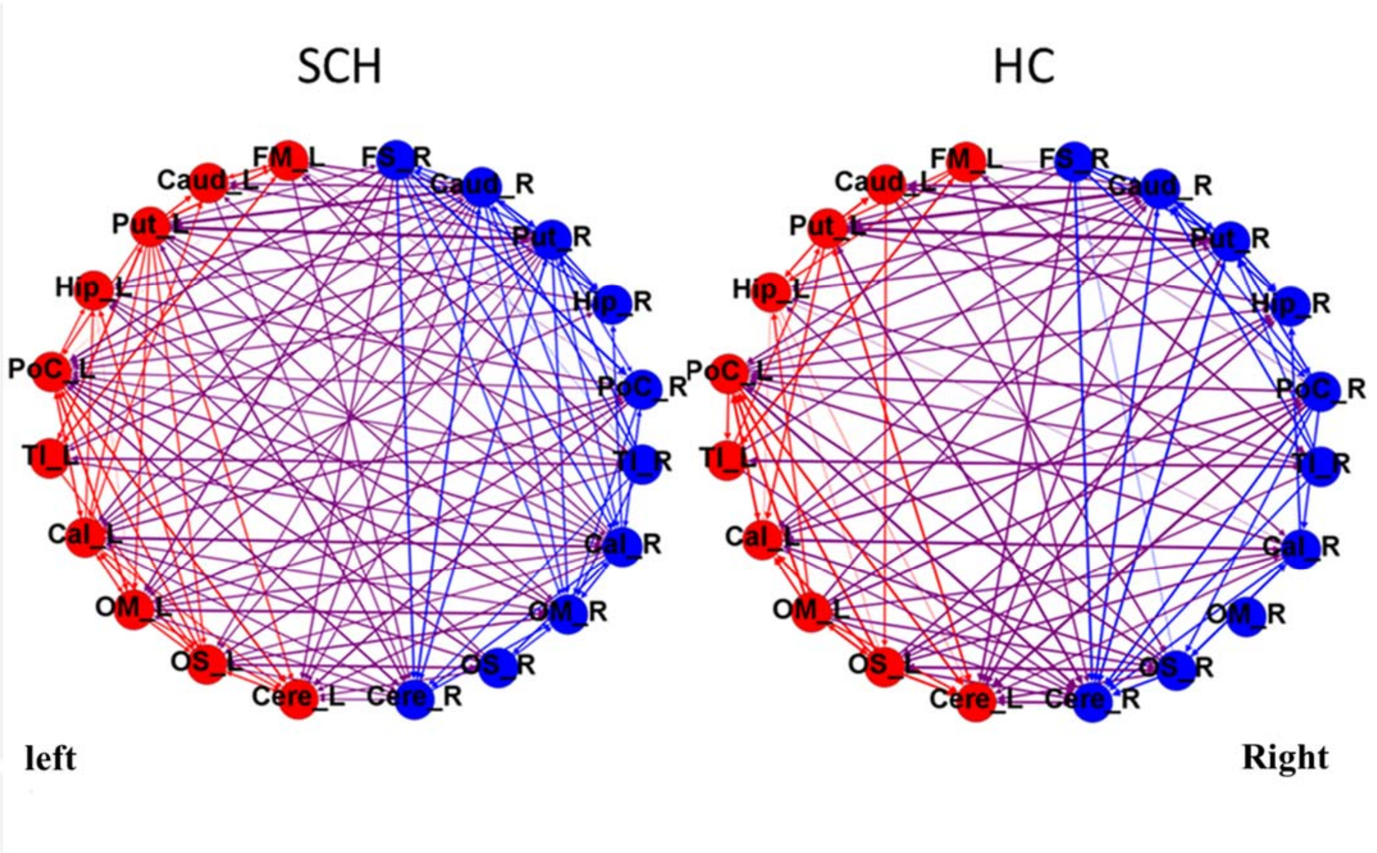

Figure S2. One sample t-test results of the effective connectivity paths among the 20 ROIs (P < 0.05, FDR corrected) (|t| value range: 2.40 - 12.85). Left brain regions are depicted in red while the right brain regions in blue. Thickness of the lines corresponds to the strength (t values) of the paths. (red line: connection within the left hemisphere; blue line: connection within the right hemisphere; purple line: the interhemisphere connection).

3. Supplementary tables

Table S1. The averaged values of magnitudes of Granger- causality interaction in schizophrenia patients (part 1)

		origin									
		IC_L	Put_L	Caud_L	Hip_L	ITG_L	MFG_L	PoC_L	Cal_L	SOG_L	MOG_L
target	IC_L		0.0407±0.0121*	0.0037±0.013	0.0352±0.0189	0.0271±0.0129	-0.0015±0.0069	0.0924±0.0152*	0.0203±0.0082*	0.0412±0.0131*	0.0342±0.0096*
	Put_L	0.0014±0.009		0.0229±0.0096	-0.0126±0.0095	0.0316±0.0076*	0.025±0.0051*	0.0111±0.0111	0.0102±0.0077	0±0.0103	0.0155±0.008
	Caud_L	0.0087±0.0209	0.0733±0.0167*		0.0351±0.0191	0.0367±0.0173	0.0294±0.010*	-0.0134±0.0157	0.033±0.012*	0.0073±0.013	0.0277±0.0121
	Hip_L	0.038±0.016	0.0271±0.0133	0.0206±0.0124		0.0126±0.0134	0.016±0.0077	0.0329±0.0128*	0.0212±0.0081*	0.0505±0.0136*	0.0092±0.0128
	ITG_L	0.0091±0.0167	0.0531±0.0153*	-0.0104±0.0308	-0.0349±0.0196		0.0478±0.01*	0.0392±0.0181	0.0069±0.0136	0.0416±0.022	-0.0203±0.0116
	MFG_L	-0.0253±0.023	0.1033±0.0299*	0.0862±0.0299*	-0.0187±0.0269	0.0476±0.0195*		-0.0773±0.0218*	0.0216±0.0121	-0.0211±0.0205	-0.0349±0.0168
	PoC_L	0.0186±0.0111	0.0287±0.0088*	0.0061±0.0087	0.0052±0.01	0.015±0.0094	-0.0001±0.0064		0.0236±0.0065*	0.0283±0.0099*	0.0313±0.0067*
	Cal_L	0.0273±0.0251	0.0977±0.0292*	0.0218±0.0298	-0.0389±0.0268	0.0409±0.0216	0.0375±0.0183	0.0995±0.0244*		0.1506±0.0297*	0.0572±0.0235*
	SOG_L	0.0415±0.0152*	0.0499±0.0164*	0.0232±0.0141	-0.0115±0.0161	0.0401±0.0143*	-0.0111±0.01	0.0454±0.0175*	0.0191±0.0091		0.0441±0.0117*
	MOG_L	0.0113±0.0185	0.0717±0.0211*	0.0124±0.0189	-0.0716±0.0249*	0.0302±0.0166	0.0167±0.0117	0.1126±0.0251*	0.0298±0.0099*	0.0435±0.0195	
	IC_R	0.015±0.0179	0.054±0.0142*	0.0286±0.0112*	-0.0161±0.0193	0.0171±0.0138	0.0237±0.007*	0.0728±0.0144*	0.0331±0.0111*	-0.0005±0.0118	0.0177±0.0097
	Put_R	0.0145±0.0106	0.1455±0.015*	0.0223±0.0107	-0.0293±0.0112*	0.027±0.0084*	0.0239±0.0066*	0.0228±0.0126	0.0069±0.0072	0.0101±0.0115	0.0088±0.0072
	Caud_	0.0121±0.011	0.1249±0.016	0.0131±0.015	-0.0207±0.012	0.0277±0.009	0.0312±0.007	0.0195±0.015	0.0106±0.008	0.0073±0.014	0.0124±0.009

	R	7	3*	1		7*	9*	4		6	8
	Hip_R	0.0187±0.0149	0.0555±0.0139*	-0.0114±0.0138	-0.025±0.0187	0.0356±0.0151	0.0074±0.0068	0.0482±0.0155*	0.041±0.0109*	0.0212±0.0163	0.0335±0.0139
	ITG_R	-0.0181±0.0188	0.0109±0.0189	0.0011±0.0179	-0.02±0.02	0.0584±0.0224*	0.0352±0.0125*	-0.0074±0.0227	-0.006±0.0103	-0.0178±0.0207	-0.0073±0.0135
	SFG_R	-0.0163±0.0122	0.0521±0.019*	0.0126±0.0102	-0.023±0.013	0.0042±0.0078	-0.0148±0.0062	-0.0009±0.0165	0.0176±0.0088	-0.0231±0.0124	0.0017±0.0103
	PoC_R	0.0096±0.012	0.0256±0.0121	-0.0008±0.0118	-0.0032±0.012	0.003±0.0103	-0.021±0.0057*	0.0216±0.0152	0.0209±0.0085*	0.0384±0.0131*	0.0317±0.0076*
	Cal_R	0.0238±0.0191	0.0683±0.0246*	0.0232±0.0195	-0.063±0.0222*	0.0599±0.017*	-0.0038±0.013	0.0757±0.0236*	0.0995±0.0176*	0.0568±0.0251	0.0343±0.0162
	SOG_R	0.009±0.0156	0.0369±0.0167	0.0196±0.0172	-0.0271±0.0172	0.0088±0.0166	-0.0145±0.0111	0.0711±0.0186*	0.0302±0.0128	0.094±0.0176*	0.0311±0.0143
	MOG_R	0.0192±0.0195	0.0818±0.0224*	0.0192±0.0197	-0.0671±0.0227*	0.0419±0.0219	0.0228±0.0135	0.0648±0.0258*	0.0477±0.0153*	0.0827±0.019*	0.0895±0.0121*

Note: The row represents “origin”, while the column represents “target”. The asterisk represents the significant causality interaction ($P < 0.05$, FDR corrected). Means ± standard errors across subjects were reported.

Table S1. The averaged values of magnitudes of Granger- causality interaction in schizophrenia patients (part 2)

		origin									
		IC_R	Put_R	Caud_R	Hip_R	ITG_R	SFG_R	PoC_R	Cal_R	SOG_R	MOG_R
target	IC_L	0.0459±0.0177*	0.0362±0.0143*	0.0431±0.0127*	0.0124±0.0152	-0.016±0.0109	0.0499±0.0122*	0.1005±0.018*	0.0321±0.0086*	0.0334±0.0109*	0.0131±0.0086
	Put_L	0.0314±0.0081*	0.1198±0.0116*	0.0958±0.0102*	-0.0056±0.0092	0.0281±0.0072*	0.0101±0.008	-0.0094±0.009	0.0123±0.0064	0.0126±0.0079	-0.0009±0.0067
	Caud_L	0.0204±0.0158	0.0652±0.0159*	0.0621±0.0131*	0.0187±0.0182	-0.0007±0.0165	-0.0197±0.0158	-0.034±0.018	0.0415±0.0129*	0.0072±0.0173	0.0074±0.0115
	Hip_L	0.0135±0.0152	0.044±0.0124*	0.0419±0.0098*	0.0141±0.013	0.0162±0.0083	0.0203±0.0097	0.061±0.0151*	0.0259±0.0101*	0.0412±0.0115*	0.0313±0.0084*
	ITG_L	0.0364±0.019	0.0486±0.0186*	0.0399±0.0178	-0.009±0.0179	0.0755±0.0142*	-0.0281±0.0171	-0.028±0.0152	0.042±0.0184	0.0599±0.026	0.0047±0.0205
	MFG_L	-0.0026±0.0215	0.0412±0.0229	0.0589±0.0217*	0.028±0.0218	0.0686±0.0219*	-0.0418±0.0202	-0.0608±0.0235*	0.0436±0.0169*	0.0155±0.0193	-0.044±0.0206
	PoC_L	0.0263±0.0103*	0.0336±0.0072*	0.025±0.0061*	0.0105±0.008	0.0191±0.0069*	0.0384±0.0066*	0.0251±0.01*	0.0268±0.0062*	0.0253±0.0072*	0.0282±0.0066*
	Cal_L	0.036±0.0216	0.0965±0.027*	0.0992±0.0247*	-0.0064±0.0249	0.0084±0.0251	0.0814±0.0196*	0.1236±0.0268*	0.1968±0.0307*	0.1335±0.0294*	0.0522±0.0218
	SOG_L	0.0359±0.0129*	0.0365±0.0147*	0.0342±0.0137*	-0.0207±0.0138	0.0016±0.0095	0.0196±0.015	0.0653±0.0182*	0.0262±0.01*	0.0676±0.0148*	0.0268±0.0107*
	MOG_L	0.0441±0.0139*	0.0601±0.0162*	0.0383±0.0137*	-0.0339±0.0186	0.0408±0.011*	0.0747±0.0142*	0.0779±0.0183*	0.0501±0.0125*	0.0172±0.0163	0.0325±0.0149
	IC_R		0.0435±0.0131*	0.0521±0.0114*	-0.0056±0.0182	0.0247±0.0097*	0.0566±0.0106*	0.0373±0.0157	0.0383±0.0095*	0.0211±0.0096	0.0187±0.0087
	Put_R	0.0308±0.0094*		0.1005±0.017*	-0.007±0.0103	0.0374±0.0077*	0.0164±0.0082	-0.0003±0.0089	0.0099±0.0067	0.0141±0.0082	0.0004±0.0075
	Caud_R	0.0362±0.0101*	0.0842±0.0216*		-0.0179±0.01	0.0394±0.0088*	0.0016±0.0107	0.0006±0.0108	0.0113±0.0078	0.011±0.0092	-0.0025±0.0101
	Hip_R	0.0299±0.015	0.0515±0.011	0.0516±0.011		0.0165±0.009	0.0404±0.010	0.0399±0.020	0.0433±0.011	0.0163±0.012	0.0035±0.009

		9	4*	2*			7*	2	9*	6	6
	ITG_R	0.0166±0.0171	0.0178±0.0198	0.0233±0.0156	-0.0383±0.0185		-0.0351±0.0139*	0.0126±0.0207	0.0169±0.013	0.0123±0.0166	-0.0276±0.0141
	SFG_R	-0.0167±0.012	0.0492±0.0145*	0.04±0.0136*	0±0.0109	0.0064±0.0088		0.0028±0.0124	0.0033±0.007	-0.02±0.0098	-0.0047±0.0087
	PoC_R	0.0094±0.0117	0.0259±0.0103*	0.012±0.0091	0.0047±0.0106	-0.0073±0.0076	0.0811±0.0108*		0.0253±0.008*	0.017±0.0094	0.0188±0.0079
	Cal_R	-0.0007±0.0164	0.0622±0.0198*	0.0576±0.0182*	-0.026±0.0194	0.0118±0.0147	0.052±0.0138*	0.059±0.0196*		0.0636±0.0207*	0.0086±0.0162
	SOG_R	0.0352±0.0146	0.0251±0.0136	0.0214±0.0136	-0.0195±0.0184	-0.003±0.0112	0.0244±0.0157	0.0394±0.0168	0.0454±0.0133*		0.0167±0.0165
	MOG_R	0.0616±0.016*	0.0735±0.0181*	0.0598±0.0178*	-0.0107±0.0205	0.0659±0.0176*	0.0532±0.0197*	0.0822±0.021*	0.0675±0.0162*	0.0515±0.0186*	

Note: The row represents "origin", while the column represents "target". The asterisk represents the significant causality interaction ($P < 0.05$, FDR corrected). Means ± standard errors across subjects were reported.

Table S2. The averaged values of magnitudes of Granger- causality interaction in healthy controls (part 1)

		origin									
		IC_L	Put_L	Caud_L	Hip_L	ITG_L	MFG_L	PoC_L	Cal_L	SOG_L	MOG_L
target	IC_L		0.0588±0.013*	-0.0004±0.0139	-0.0424±0.0143*	0.0115±0.0102	-0.0052±0.0075	0.0853±0.0146*	0.0062±0.0071	0.0485±0.0087*	0.028±0.007*
	Put_L	0.033±0.01*		0.0148±0.0126	0.0027±0.0126	0.0193±0.0075*	0.0206±0.0075*	0.0092±0.0087	0.0004±0.0047	-0.0027±0.0083	0.0081±0.0054
	Caud_L	0.0037±0.0137	0.0901±0.0184*		-0.005±0.0162	0.0034±0.012	0.0179±0.0093	-0.0072±0.0158	0.0098±0.0062	-0.0031±0.0075	0.0093±0.0061
	Hip_L	0.0169±0.0099	0.0348±0.0105*	-0.0011±0.0117		-0.0045±0.0094	0.0198±0.0072*	0.0151±0.0097	-0.0027±0.0048	0.0051±0.0067	0.0149±0.0052*
	ITG_L	-0.0067±0.0198	0.055±0.0248	0.0367±0.0251	-0.02±0.024		0.0765±0.0151*	0.0707±0.0212*	0.0158±0.011	0.0189±0.0172	0.0211±0.0117
	MFG_L	-0.0507±0.0216	0.0484±0.0212	0.0307±0.0239	-0.0504±0.0243	0.0469±0.0164*		-0.0461±0.0202	0.0072±0.0096	-0.011±0.0138	0.0082±0.0101
	PoC_L	-0.0024±0.0165	0.0223±0.0122	0.0227±0.0114	-0.0223±0.0127	0.0173±0.01	-0.0072±0.0072		0.0277±0.0055*	0.0579±0.0088*	0.0428±0.0062*
	Cal_L	-0.0676±0.039	0.1496±0.0396*	-0.0345±0.048	-0.1405±0.0376*	0.0575±0.0269	0.0339±0.0206	0.1174±0.0412*		0.2233±0.0408*	0.0948±0.022*
	SOG_L	-0.0384±0.0193	0.0059±0.0144	0.051±0.0208*	-0.0614±0.0188*	0.0284±0.0122	0.0095±0.0104	0.0431±0.0198	0.0192±0.0108		0.0612±0.012*
	MOG_L	-0.0663±0.0303	0.0514±0.0242	0.0466±0.0235	-0.0917±0.029*	-0.0064±0.018	0.0205±0.0135	0.0232±0.0255	-0.0092±0.0134	0.057±0.0208*	
	IC_R	0.074±0.0154*	0.0704±0.0124*	0.0185±0.0135*	-0.0166±0.0135	0.0262±0.0096*	0.0276±0.0086*	0.0874±0.0118*	0.0164±0.0068*	0.0478±0.0101*	0.0318±0.0079*
	Put_R	0.0246±0.0099*	0.13±0.0138*	0.0391±0.0115*	-0.0205±0.0118	0.0098±0.0099	0.0072±0.0066	0.0255±0.0089*	-0.0003±0.0052	0.005±0.0085	0.0118±0.0057
	Caud_R	0.0252±0.0106	0.1312±0.015*	0.0477±0.0125	-0.0277±0.0136	0.0294±0.0101*	0.0225±0.0072*	0.0358±0.0098*	0.0064±0.0051	0.0127±0.0087	0.0204±0.0061*
	Hip_R	0.007±0.0126	0.0579±0.0135*	0.0132±0.0124	-0.0009±0.017	0.0171±0.0103	0.0126±0.0061	0.0435±0.0122*	0.0155±0.0047*	0.0296±0.0077*	0.0272±0.0056*

	ITG_R	-0.0465±0.0208	-0.0335±0.0179	0.0119±0.0204	-0.0082±0.0232	0.0592±0.0233*	0.052±0.0111*	0.027±0.0195	0.0096±0.0089	0.027±0.017	0.0253±0.0118
	SFG_R	-0.0327±0.0152	0.027±0.0146	0.0132±0.015	-0.0273±0.0156	0±0.0112	-0.0422±0.0093*	-0.0738±0.0147*	-0.0114±0.0068	-0.024±0.01	-0.012±0.0063
	PoC_R	-0.0084±0.0174	0.02±0.0172	0.033±0.0177	-0.0374±0.016	-0.0086±0.01	-0.0202±0.008*	0.0113±0.0185	-0.0019±0.0062	0.0373±0.0118*	0.0326±0.0075*
	Cal_R	-0.1164±0.0352*	0.0602±0.0279	0.0842±0.0292*	-0.0979±0.0278*	0.0315±0.0254	0.0059±0.0139	0.0462±0.0338	0.0686±0.0211*	0.1008±0.0241*	0.05±0.0143*
	SOG_R	-0.0608±0.0221*	0.023±0.018	0.0858±0.023*	-0.0212±0.0242	0.0135±0.0164	-0.016±0.0107	0.0402±0.0221	0.0244±0.0122	0.1191±0.0246*	0.0525±0.012*
	MOG_R	-0.0441±0.0303	0.0691±0.0222*	0.0589±0.029	-0.0483±0.032	0.0208±0.0213	0.0105±0.0126	0.083±0.0276*	0.0124±0.0162	0.139±0.0338*	0.1405±0.0262*

Note: The row represents "origin", while the column represents "target". The asterisk represents the significant causality interaction ($P < 0.05$, FDR corrected). Means ± standard errors across subjects were reported.

Table S2. The averaged values of magnitudes of Granger- causality interaction in healthy controls (part 2)

		origin									
		IC_R	Put_R	Caud_R	Hip_R	ITG_R	SFG_R	PoC_R	Cal_R	SOG_R	MOG_R
target	IC_L	0.0628±0.0153*	0.0582±0.0123*	0.0461±0.0119*	-0.0244±0.0122	0.0076±0.0086	0.0484±0.0097*	0.1064±0.0131*	0.0178±0.0071*	0.0439±0.0094*	0.0281±0.0078*
	Put_L	0.0282±0.009*	0.1364±0.0105*	0.1026±0.0094*	0.01±0.0099	0.0065±0.0082	0.0273±0.0089*	0.0108±0.0073	-0.0014±0.005	-0.0002±0.0061	-0.0021±0.0056
	Caud_L	0.0196±0.0127	0.081±0.0123*	0.0766±0.0111*	0.0206±0.0137	-0.002±0.0118	0.0147±0.0089	0.0111±0.0142	0.0149±0.0073	0.007±0.0076	0.0093±0.0072
	Hip_L	0.0151±0.0095	0.0316±0.0094*	0.0275±0.0092*	0.011±0.0094	0.0184±0.0067*	0.0112±0.0069	0.0113±0.0089	0.0093±0.006	0.0151±0.006*	0.0014±0.0045
	ITG_L	-0.0224±0.0186	0.0099±0.0207	0.0235±0.0183	0.0017±0.0249	0.105±0.0174*	0.022±0.0137	0.0318±0.0181	0.0054±0.0111	0.0086±0.0148	0.0246±0.011
	MFG_L	-0.011±0.0191	-0.0098±0.0167	0.034±0.0185	-0.028±0.0205	0.0613±0.0231*	-0.0391±0.018	-0.0297±0.0161	0.001±0.0094	-0.006±0.0118	0.002±0.0094
	PoC_L	0.0155±0.0148	0.0193±0.0122	0.0074±0.011	0.0113±0.0113	0.0212±0.0076*	0.0258±0.0079*	0.045±0.0093*	0.0253±0.0056*	0.0409±0.0074*	0.0414±0.0072*
	Cal_L	-0.0227±0.0329	0.1223±0.0459*	0.1095±0.0407*	-0.0822±0.0384	0.0444±0.0263	0.0415±0.0377	0.1921±0.0369	0.2679±0.0366*	0.1662±0.0343*	0.0672±0.0244*
	SOG_L	-0.0094±0.0167	0.0194±0.0152	0.0004±0.0146	-0.0429±0.02	0.0335±0.0126*	0.0095±0.0112	0.0751±0.0149	0.0315±0.013*	0.0898±0.0152*	0.0612±0.0115*
	MOG_L	-0.0639±0.0242*	-0.0002±0.0171	-0.0206±0.0194	-0.0874±0.0277*	0.0319±0.0206	0.0297±0.0195	0.0889±0.0243	-0.0016±0.0143*	0.0227±0.0172	0.0655±0.0189
	IC_R		0.0689±0.0156*	0.0674±0.0149*	0.0162±0.015	0.0335±0.0105*	0.0543±0.0099*	0.0639±0.013*	0.0285±0.008*	0.0355±0.0073*	0.039±0.0094*
	Put_R	0.0316±0.0115*		0.0646±0.0155*	0.0036±0.0104	0.0206±0.0081*	0.0404±0.009*	0.0221±0.0073*	-0.0052±0.0051	0.0077±0.0066	0.0035±0.005
	Caud_R	0.0323±0.0123*	0.1029±0.0231*		0.0055±0.0109	0.0389±0.0087*	0.0439±0.0088*	0.019±0.0074*	-0.0001±0.0053	0.0116±0.007	0.0127±0.0063

	Hip_R	0.0249±0.0114	0.0642±0.0125*	0.0587±0.0124*		0.0082±0.0094	0.0174±0.0092	0.0292±0.0126	0.0232±0.0055*	0.0165±0.0069	0.0106±0.0064
	ITG_R	-0.0169±0.014	-0.012±0.0138	-0.0097±0.0168	-0.0476±0.0206		-0.0219±0.0152	0.0202±0.0173	0.0252±0.0112	0.0165±0.0096	0.0233±0.0112
	SFG_R	-0.0289±0.0143	0.0311±0.0134	0.0251±0.0119	-0.0094±0.0159	-0.0018±0.0091		-0.0264±0.0127	-0.0125±0.0069	-0.0217±0.0072*	-0.0219±0.0073*
	PoC_R	-0.0318±0.0148	0.0424±0.0142*	0.0155±0.0128	-0.0209±0.0152	0.0076±0.0097	0.0523±0.012*		-0.0038±0.006'	0.0199±0.0095	0.0171±0.0091
	Cal_R	-0.0842±0.0355	0.0301±0.0316	0.0122±0.0293	-0.0755±0.024*	0.0155±0.0209	0.0363±0.0174	0.0935±0.0268*		0.087±0.0208*	0.0253±0.0137
	SOG_R	-0.0341±0.0238	0.0129±0.0184	-0.013±0.017	-0.0531±0.0234	0.0153±0.0122	-0.0246±0.0115	0.0555±0.0164*	0.042±0.012*		0.0652±0.0129*
	MOG_R	-0.028±0.0254	0.0405±0.0205	0.0151±0.0195	-0.129±0.0265*	0.0386±0.0221	0.006±0.0192	0.0959±0.025*	0.022±0.017	0.0884±0.0219*	

Note: The row represents “origin”, while the column represents “target”. The asterisk represents the significant causality interaction ($P < 0.05$, FDR corrected). Means ± standard errors across subjects were reported.
